# Novel hepatic microRNAs upregulated in human nonalcoholic fatty liver disease

**DOI:** 10.14814/phy2.12661

**Published:** 2016-01-05

**Authors:** Jarkko Soronen, Hannele Yki‐Järvinen, You Zhou, Sanja Sädevirta, Antti‐Pekka Sarin, Marja Leivonen, Ksenia Sevastianova, Julia Perttilä, Pirkka‐Pekka Laurila, Alexander Sigruener, Gerd Schmitz, Vesa M. Olkkonen

**Affiliations:** ^1^ Genomics and Biomarkers UnitNational Institute for Health and WelfareBiomedicumHelsinkiFinland; ^2^ Minerva Foundation Institute for Medical ResearchHelsinkiFinland; ^3^ Department of MedicineDivision of DiabetesUniversity of HelsinkiHelsinkiFinland; ^4^ Institute for Molecular Medicine Finland FIMMUniversity of HelsinkiHelsinkiFinland; ^5^ Department of SurgeryHelsinki University Central HospitalHelsinkiFinland; ^6^ Institute of Clinical Chemistry and Laboratory MedicineRegensburg University Medical CenterRegensburgGermany; ^7^ Institute of Biomedicine, AnatomyUniversity of HelsinkiHelsinkiFinland

**Keywords:** Hepatocyte, immortalized human hepatocytes, liver biopsy, microarray, microRNA, nonalcoholic fatty liver disease

## Abstract

MicroRNAs (miRNAs) control gene expression by reducing mRNA stability and translation. We aimed to identify alterations in human liver miRNA expression/function in nonalcoholic fatty liver disease (NAFLD). Subjects with the highest (median liver fat 30%, *n* = 15) and lowest (0%, *n* = 15) liver fat content were selected from >100 obese patients for miRNA profiling of liver biopsies on microarrays carrying probes for 1438 human miRNAs (a cross‐sectional study). Target mRNAs and pathways were predicted for the miRNAs most significantly upregulated in NAFLD, their cell‐type‐specific expression was investigated by quantitative PCR (qPCR), and the transcriptome of immortalized human hepatocytes (IHH) transfected with the miRNA with the highest number of predicted targets, miR‐576‐5p, was studied. The screen revealed 42 miRNAs up‐ and two downregulated in the NAFLD as compared to non‐NAFLD liver. The miRNAs differing most significantly between the groups, miR‐103a‐2*, miR‐106b, miR‐576‐5p, miRPlus‐I137*, miR‐892a, miR‐1282, miR‐3663‐5p, and miR‐3924, were all upregulated in NAFLD liver. Target pathways predicted for these miRNAs included ones involved in cancer, metabolic regulation, insulin signaling, and inflammation. Consistent transcriptome changes were observed in IHH transfected with miR‐576‐5p, and western analysis revealed a marked reduction of the RAC1 protein belonging to several miR‐576‐5p target pathways. To conclude, we identified 44 miRNAs differentially expressed in NAFLD versus non‐NAFLD liver, 42 of these being novel in the context of NAFLD. The study demonstrates that by applying a novel study set‐up and a broad‐coverage array platform one can reveal a wealth of previously undiscovered miRNA dysregulation in metabolic disease.

## Introduction

Nonalcoholic fatty liver disease (NAFLD) is a disorder characterized by hepatic fat accumulation due to nonalcoholic causes (Perry et al. 2014). This burgeoning health problem is closely associated with all features of the metabolic syndrome (Yki‐Järvinen 2014), and its prevalence increases rapidly worldwide with the expanding epidemic of obesity (Bellentani et al. 2010). NAFLD predicts type 2 diabetes, cardiovascular diseases, nonalcoholic steatohepatitis, and hepatocellular carcinoma (White et al. 2012; Yki‐Järvinen 2014).

MicroRNAs (miRNAs) are short (19–23 nt) noncoding RNA molecules that confer a crucial layer of regulation of gene expression via mRNA destabilization, translational repression, or activation of transcription/translation (Vasudevan et al. 2007; Selbach et al. 2008). Altered miRNA expression is associated with a spectrum of diseases including metabolic disorders (Rottiers and Naar 2012; Williams and Mitchell 2012). The role of miRNA‐mediated gene regulation in liver function and diseases has gained increasing attention (Ferreira et al. 2014). Studies on human NAFLD (Cheung et al. 2008; Vinciguerra et al. 2009; Cazanave et al. 2011; Kida et al. 2011; Trajkovski et al. 2011; Min et al. 2012; Ogawa et al. 2012; Castro et al. 2013; Li et al. 2014a; Pirola et al. 2014) have identified approximately 50 miRNAs dysregulated in NAFLD liver, and the current view is that they critically contribute to the development and progression of the disease. A number of the dysregulated miRNAs are involved in hepatic fatty acid synthesis, uptake, storage as triglycerides, or β‐oxidation. In a pioneering screen Cheung et al. (2008) compared hepatic miRNA profiles in 25 human subjects with metabolic syndrome and advanced NAFLD (nonalcoholic steatohepatitis, NASH) and 25 healthy controls, by using microarrays carrying probes for 474 human miRNAs. They found 46 miRNAs differentially expressed, 23 up‐ and 23 downregulated in the NASH group. Among the differentially expressed miRNAs there was miR‐122 (reduced in the NASH group), a major hepatic species that negatively regulates hepatic lipogenesis (Fukuhara and Matsuura 2013), and a number of miRNAs with functions in cell growth and differentiation, apoptosis, and inflammation. MiR‐34a, which regulates the sirtuin 1/p53 pathway inhibiting the synthesis and stimulating the β‐oxidation of fatty acids, was shown to be upregulated in subjects with NAFLD, its activity correlating with disease severity (Castro et al. 2013). Peroxisome proliferator‐activated receptor α (PPARα), a transcription factor with a pivotal role in hepatic lipid metabolism, oxidative stress, inflammatory response, and fibrogenesis, was shown to be targeted in hepatoma cells by miR‐21, and correlated negatively with this miRNA species in human liver (Kida et al. 2011). miR‐296‐5p, which modulates lipoapoptosis, was demonstrated to be reduced in the liver of subjects with advanced NAFLD as compared to patients with simple steatosis or healthy controls (Cazanave et al. 2011).

Besides lipid metabolism, control of hepatic insulin sensitivity, inflammation, and fibrosis by miRNAs have received increasing attention. Trajkovski et al. (2011) identified in mice miR‐103 and ‐107 as species upregulated in NAFLD liver, confirmed this result in a human cohort, and provided evidence for roles of these miRNAs as negative regulators of insulin sensitivity. miR‐21, which is upregulated in steatotic liver of rodents and humans is a potent inhibitor of the phosphatase and tensin homolog (PTEN) (Vinciguerra et al. 2009), a phosphoinositide phosphatase that dephosphorylates PtdIns(3‐5)P_3_, a key mediator of insulin signaling. Inhibition of PTEN expression by miR‐21 leads to a constitutive activation of AKT that desensitizes the cells to further insulin action (Vinciguerra et al. 2008).

The miRNAs associated with hepatic inflammation and fibrosis have mainly been studied in patients infected by hepatitis C virus (Jiang et al. 2014; Sarma et al. 2014). However, the drastic reduction of miR‐122 (Cheung et al. 2008) in subjects with NASH may be causally related to the hepatic inflammation; Knock‐out of this miRNA in mouse resulted in steatohepatitis, liver fibrosis, and upregulation of the proinflammatory cytokines interleukin‐6 (IL‐6), tumor necrosis factor‐α (TNF‐α), and the chemokine CCL2, as well as the recruitment of immune cells in the liver (Wen and Friedman 2012). Transforming growth factor‐β (TGF‐β), secreted by sinusoidal endothelial cells, Kupffer cells, activated stellate cells (HSC) or damaged hepatocytes, is the most important activator of HSC transdifferentiation into myofibroblasts in liver fibrosis (Dooley and ten Dijke 2012). Several miRNAs have been suggested to modulate TGF‐β signaling in fibrotic human liver. These include miR‐19b, which targets TGF‐β receptor II and is reduced in fibrotic liver (Lakner et al. 2012), and miR‐21, which is drastically upregulated in fibrotic liver and inhibits SMAD7, a negative regulator of TGF‐β signaling (Zhang et al. 2013).

These and other studies have provided clues of miRNA function in the etiology of NAFLD. However, they have addressed miRNA expression in steatohepatitis or advanced NAFLD with prominent fibrosis, focused on the expression/function of individual miRNAs/miRNA families, or employed mouse models or cultured hepatocytes (recently reviewed in Sobolewski et al. 2015), while up‐to‐date tools capturing most of the human miRNome have not been employed for profiling of the NAFLD liver. In this study, we aimed to extensively characterize changes in liver miRNA expression in NAFLD on a platform detecting 1438 human miRNAs.

## Materials and Methods

### Subjects and study design

Subjects were recruited among those undergoing a laparoscopic gastric bypass or sleeve gastrectomy in Peijas Hospital of the Hospital District of Helsinki and Uusimaa, Finland. The inclusion criteria were as follows: (1) age 18–65 years; (2) no known acute or chronic disease except for obesity or obesity related diseases such as type 2 diabetes, NAFLD, cardiovascular diseases and/or hyperlipidemia, based on history, physical examination, standard laboratory tests, and electrocardiogram. Exclusion criteria were as follows: (1) excessive use of alcohol (over 20 g/day), (2) use of hepatotoxic medications or herbal products, and (3) pregnancy or lactation. The subjects were studied in the morning after an overnight (10–12 h) fast 1–2 weeks prior to surgery. Weight, height, waist, and hip circumferences were recorded as described previously (Kotronen et al. 2007). Blood samples were taken for measurement of complete blood cell count and concentrations of alanine aminotransferase (ALT), aspartate aminotransferase (AST), alkaline phosphatase (ALP), high‐density lipoprotein (HDL) and low‐density lipoprotein (LDL) cholesterol, triglycerides, glucose, C‐peptide, and insulin, as previously described (Kotronen et al. 2007). Of over 100 consecutive subjects recruited, we chose for miRNA profiling 15 with the lowest and 15 with the highest degree of macrovesicular hepatic steatosis.

### Statistical analyses of clinical parameters

All clinical data were tested for normality of distribution using a Kolmogorov–Smirnov test. Normally distributed data are shown as means ± SEM and non‐normally distributed data as median (25–75% percentile). Demographic and clinical parameters among the study groups were compared using Fisher's exact test or Chi‐square test for categorical variables, and unpaired *t*‐test or Mann–Whitney test for continuous variables. Two‐tailed *P* < 0.05 were considered statistically significant. The calculations were performed using GraphPad Prism version 4.03 for Windows (GraphPad Software Inc., San Diego, CA), Microsoft Office Excel 2007 for Windows (Microsoft, Redmond, WA) and IBM SPSS Statistics 20 (IBM Corporation, Chicago, IL).

### Ethics

The nature and potential risks of the study were explained to all subjects prior to obtaining their written informed consent. The protocol was approved by the Medicinal Ethics Committee of the Helsinki and Uusimaa Hospital District (permission 28/13/03/02/2010).

### Liver biopsies and their histopathological assessment

Wedge biopsies of the liver were taken at the time of surgery. One‐half of the biopsy specimen was sent to the pathologist for histopathological assessment, and the rest was immediately frozen in liquid nitrogen. Triglyceride content (% of hepatocytes with macrovesicular and microvesicular steatosis), stage, grade, and presence of necroinflammation were determined under light microscope by a liver pathologist as previously described (Kleiner et al. 2005).

### miRNA profiling of liver tissue

Total RNA (300 ng) from both sample and reference was labeled with Hy3^™^ and Hy5^™^ fluorescent labels, respectively, using the miRCURY LNA^™^ microRNA Hi‐Power Labeling Kit, Hy3^™^/Hy5^™^ (Exiqon, Vedbaek, Denmark) following the procedure described by the manufacturer. The Hy3^™^‐labeled samples and Hy5^™^‐labeled reference RNA samples were mixed pair‐wise and hybridized to the 6th Gen miRCURY LNA^™^ microRNA Array. The hybridization was performed according to the miRCURY LNA^™^ microRNA Array Instruction manual using a Tecan (Männedorf, Switzerland) HS4800^™^ hybridization station. The array slides were scanned using the Agilent G2565BA Microarray Scanner System (Agilent, Santa Clara, CA) and the image analysis was carried out using the ImaGene^®^ 9 software (Exiqon). The quantified signals were background corrected (Normexp with offset value 10) and normalized using the global Locally Weighted Scatterplot Smoothing regression algorithm.

Background threshold was calculated for each microarray slide. miRNAs with intensities above this threshold in less than 20% of the samples were removed from the final dataset used for the expression analysis. Expression analysis was done by using R/Bioconductor and limma package. Statistical significance was assessed by Student's *t*‐test and *P*‐values were adjusted for multiple testing by Benjamini–Hochberg (BH) and Bonferroni correction methods. The data are deposited at http://www.ncbi.nlm.nih.gov/geo/ (access code pending).

### Quantitative reverse transcription‐PCR (qPCR)

Total RNA (1.0 μg) from the liver of 10 subjects with the lowest and 10 with the highest liver fat content was reverse transcribed using the Exiqon Universal cDNA Synthesis Kit. qPCR was performed on LightCycler^®^480 II (Roche, Basel, Switzerland) using miRNA‐specific primers sets (Exiqon) and a SYBR‐Green kit (Roche). Expression data were normalized using SNORD38B. Presence of miRNAs of interest in miRNA preparations from primary human hepatocytes, stellate cells, sinusoid endothelial cells (ScienCell Research Laboratories, Carlsbad, CA), and in total RNA from human macrophages was similarly assessed.

### Cell culture

Immortalized human hepatocytes (IHH) (Samanez et al. 2012) were grown in William's E medium (Gibco/Life Technologies, Carlsbad, CA), 10% fetal bovine serum (FBS), 2 mmol/L l‐glutamine, 100 U/mL penicillin and 100 μg/mL streptomycin, 100 nmol/L (20 mU/mL) insulin and 50 nmol/L dexamethasone (Sigma‐Aldrich, St. Louis, MO), on CellBIND^®^ plates (Corning, Corning, NY). Human monocytes derived from healthy donors were isolated from fresh buffy coats supplied by the Finnish Red Cross Blood Service. Monocytes were differentiated into macrophages in Macrophage SFM medium (Gibco/Life Technologies, Grand Island, NY) using macrophage colony‐stimulating factor (M‐CSF; 50 ng/mL; Nordic Biosite, Täby, Sweden).

### miRNA transfection

Immortalized human hepatocytes were transfected for 48 h using DharmaFect1 (Dharmacon, Lafayette, CO) with 100 nmol/L miR‐576‐5p mimic or nontargeting control (miScript miRNA Mimic, Qiagen, Valencia, CA; *n* = 4 for each group) in serum‐free Dulbecco's MEM (Gibco/Life Technologies), 2% fatty acid‐free BSA, 2 mmol/L glutamine, and 1 mmol/L glucose.

### Transcript profiling of miRNA mimic transfected IHH

For gene expression analysis Agilent 4 × 44K microarrays (014850) were used. 300 ng of total RNA was labeled with Cy3 using the Agilent Quick‐Amp Labeling Kit‐1 color, according to the manufacturer′s instructions. For hybridization, the Agilent Gene Expression Hybridisation Kit was used. The arrays were scanned with Agilent G2565CA Microarray Scanner. The resulting TIFF files were processed with Agilent Feature Extraction software 10.7. The raw data generated were evaluated using ChipInspector software (Genomatix, Munich, Germany). The microarray expression data were analyzed using the R/Bioconductor through a graphical user interface, Chipster (v1.4.3, CSC, Finland, http://chipster.csc.fi/). Backround was removed using Norexp method with background offset of 50. Between array normalization was carried out with quantile normalization method. The data are deposited at http://www.ncbi.nlm.nih.gov/geo/ (access code pending).

### Bioinformatic analyses

Putative miRNA targets were identified with nine algorithms: RNA22, miRanda, miRDB, TargetScan, RNAhybrid, PITA, PICTAR, Diana‐microT, and miRWalk. Targets predicted by a minimum of three algorithms were subjected to Ingenuity Pathways Analysis (Ingenuity Systems, Inc., Redwood City, CA). Enrichment of genes in pathways was assessed in comparison with a reference set in the Ingenuity knowledge base. Fisher's exact test was used to determine the significance of pathways.

### Western blotting

Anti‐RAC1 was from BD Biosciences (San Jose, CA), anti‐insulin receptor substrate 2 (IRS‐2) from Santa Cruz Biotechnology (Dallas, TX), anti‐insulin‐like growth factor 2 mRNA‐binding protein 1 (IGF2BP1) from Cell Signaling Technology (Danvers, MA), and anti‐β‐actin from Sigma‐Aldrich. Liver biopsy homogenate or IHH proteins were separated on 8, 10, or 12% Laemmli gels and transferred onto Protran^®^ nitrocellulose (Fisher Scientific, Waltham, MA). Bound primary antibodies were detected with HRP conjugates (Jackson ImmunoResearch, West Grove, PA) and visualized by enhanced chemiluminescence (Thermo Scientific/Pierce, Rockford, IL). Signals were quantified by densitometry and normalized for β‐actin.

## Results

### Characteristics of the study subjects

Clinical and biochemical characteristics of the study subjects are summarized in Table 1. The subjects in the NAFLD group (*n* = 15) had a median macroscopic liver fat content of 30 (20–40)% and those in the non‐NAFLD group (*n* = 15) 0 (0–10)%. In addition to the histological liver parameters, the NAFLD group displayed significantly higher body weight, BMI, fasting plasma glucose, serum insulin, C‐peptide, triglycerides, ALT, and ALP than the non‐NAFLD group. The grade of hepatic steatosis and the stage of fibrosis scored according to Kleiner et al. (2005) varied in the NAFLD group from 0 to 3, and necroinflammation from 0 to 2 (Table 1). None of the subjects suffered from severe liver fibrosis. In the non‐NAFLD group all histological scores were 0. The study subjects in the two groups were comparable with respect to age and gender.

**Table 1:** Characteristics of the study subjects

	NAFLD group	Non‐NAFLD group	*P*‐value
Gender (males/females)	6/9	4/11	0.70
Age (years)	49.2 ± 2.4	46.5 ± 2.6	0.45
Body composition			
Weight (kg)	140.8 ± 5.7	119.2 ± 4.3	0.005
Body mass index (kg/m²)	48.4 ± 1.4	43.3 ± 1.4	0.018
Liver histology			
Macrosteatosis (%)	30 (20–40)	0 (0–10)	<0.0001
Microsteatosis (%)	40 (20–50)	0 (0–5)	<0.0001
Stage of fibrosis (0–4)	8/4/2/1/0^a^	15/0/0/0/0	0.027
Grade of steatosis (0–3)	7/5/2/1^a^	15/0/0/0	0.012
Necroinflammation (0–2)	8/5/2^a^	15/0/0	0.010
Biochemical parameters (fasting)			
fP‐glucose (mmol/L)	6.1 ± 0.2	5.3 ± 0.2	0.012
fS‐insulin (mU/L)	22.7 ± 0.5	7.2 ± 0.7	<0.0001
fS‐C‐peptide (nmol/L)	1.44 ± 0.07	0.81 ± 0.07	<0.0001
fS‐LDL cholesterol (mmol/L)	2.8 ± 0.2	2.6 ± 0.3	0.51
fS‐HDL cholesterol (mmol/L)	1.14 ± 0.05	1.19 ± 0.09	0.67
fS‐triglycerides (mmol/L)	1.53 ± 0.12	1.16 ± 0.11	0.03
fS‐ALT (U/L)	54 (34–70)	22 (18–32)	0.0006
fS‐AST (U/L)	33 (29–49)	25 (23–38)	0.06
fS‐ALP (U/L)	79 ± 5	64 ± 4	0.03

Data are shown as mean ± SEM or median (25–75% percentile), as appropriate.

f, fasting; P, plasma; S, serum; LDL, low‐density lipoprotein; HDL, high‐density lipoprotein; ALT, alanine aminotranferase; AST, aspartate aminotranferase; ALP, alkaline phosphatase.

a The numbers separated with “/” indicate the number of subjects in each grading group, the first number corresponding to grade 0 (no fibrosis, steatosis, or necroinflammation) and the subsequent ones increasing grades of each variable.

### MicroRNAs expressed differentially in the NAFLD versus non‐NAFLD subjects

**Figure 1:**
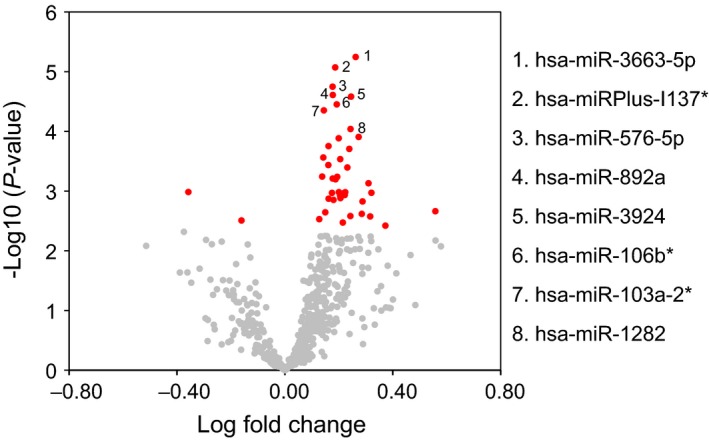
Volcano plot presentation of miRNA expression in liver biopsies of NAFLD versus non‐NAFLD subjects. The 44 microRNAs showing statistically significant (BH corrected *P* < 0.05) difference between the two groups are highlighted in red. The eight miRNAs most significantly (Bonferroni corrected *P* < 0.05) upregulated in NAFLD subjects are indicated with numbers 1–8 and identified on the right.

**Figure 2:**
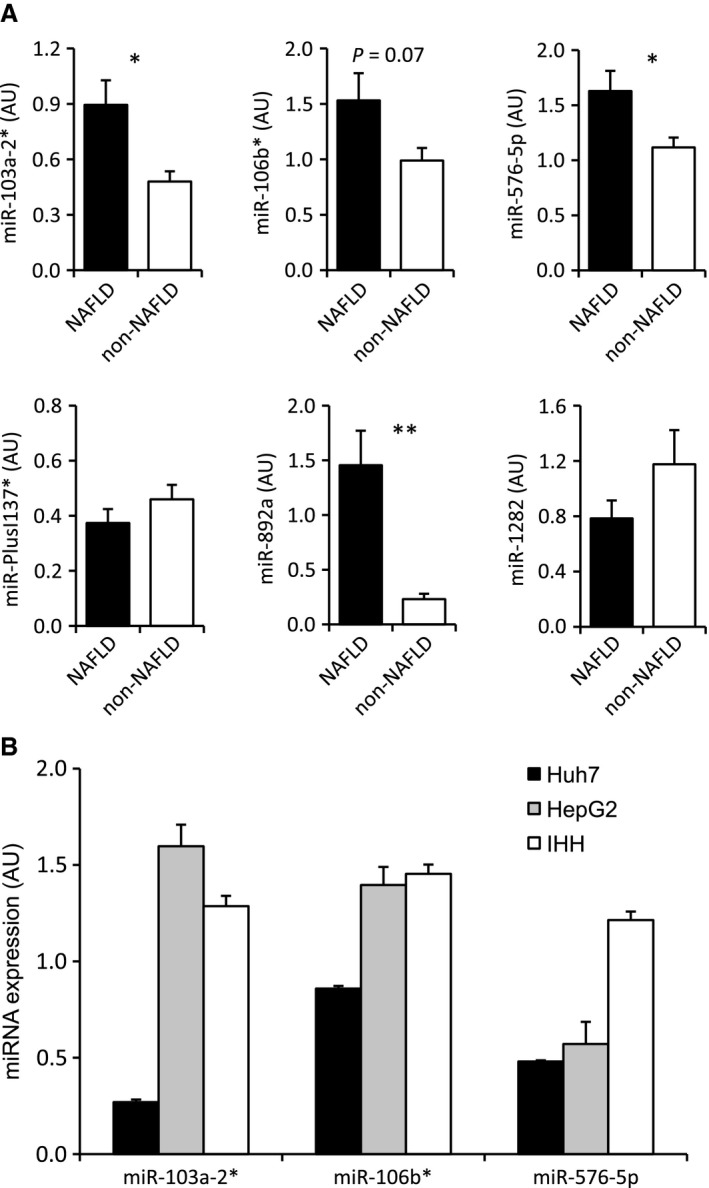
qPCR analysis of microRNA expression in liver biopsies and cultured human hepatocyte models. (A) Quantification of the indicated miRNAs in NAFLD (*n* = 10) versus non‐NAFLD (*n* = 10) biopsies. The data are normalized to SNORD38B and presented as mean ± SEM; *P < 0.05, **P < 0.01. (B) Quantification of miR‐103a‐2*, miR‐106b*, and miR‐576‐5p in the human hepatocyte models Huh7, HepG2, and IHH. The data represent mean ± SEM (*n* = 3); AU, arbitrary units.

MicroRNA profiling of liver biopsies from the NAFLD and non‐NAFLD groups was carried out on a platform with probes for 1438 human miRNAs. Between 299 and 389 miRNAs were detectable in the biopsy specimens. In the NAFLD group, 42 miRNAs were after BH multiple test correction significantly up‐ and two downregulated as compared to non‐NAFLD subjects (Fig. 1, Table 2). In further analysis, we focused on the eight miRNAs (miR‐103a‐2*, miR‐106b, miR‐576‐5p, miRPlus‐I137*, miR‐892a, miR‐1282, miR‐3663‐5p, miR‐3924, all upregulated in NAFLD) differing most significantly between the two study groups (*P* < 0.05 after Bonferroni correction). The fold change of their upregulation was between 1.11 (miR‐103a‐2*) and 1.20 miR‐3663‐5p). The functions of these miRNAs are poorly known (Table 3), and none of them have previously been associated with NAFLD. Of the eight miRNAs, six (miR‐103a‐2*, miR‐106b*, miR‐576‐5p, miRPlus‐I137*, miR‐892a, and miR‐1282) were detectable by Exiqon qPCR tools in the liver biopsies (Table 3). The first three were present in RNA from primary human hepatocytes, stellate cells, sinusoid endothelial cells, and macrophages, whereas miRPlus‐I137* was only detectable in hepatocytes and stellate cells, and miR‐892a and miR‐1282 in RNA from primary human macrophages (Table 3). Of the six miRNAs detectable in liver biopsies by qPCR, elevated expression of miR‐103a‐2*, miR‐106b*, miR‐576‐5p, and miR‐892a in NAFLD liver was confirmed by qPCR (Fig. 2A), whereas the upregulation of miRPlus‐I137* and miR‐1282 could not be validated with the available tools. The three hepatocyte miRNAs validated to be induced in NAFLD (miR‐103a‐2*, miR‐106b*, miR‐576‐5p) were present in cultured human hepatocyte models, the hepatoma cell line HuH7, the hepatocellular carcinoma line HepG2, and immortalized human hepatocytes, IHH (Fig. 2B), identifying them as feasible targets of functional analysis in cultured hepatocytes.

**Table 2:** MicroRNAs expressed differentially between the NAFLD and non‐NAFLD groups

Numbers	miRNA	*P*‐value			Fold change
		Nominal	BH	Bonferroni	
1	hsa‐miR‐3663‐5p	0.0000	0.003	0.003	1.20
2	hsa‐miRPlus‐I137*	0.0000	0.002	0.004	1.14
3	hsa‐miR‐576‐5p	0.0000	0.003	0.009	1.13
4	hsa‐miR‐892a	0.0000	0.003	0.012	1.13
5	hsa‐miR‐3924	0.0000	0.003	0.013	1.19
6	hsa‐miR‐106b*	0.0000	0.003	0.017	1.14
7	hsa‐miR‐103a‐2*	0.0000	0.003	0.022	1.11
8	hsa‐miR‐1282	0.0001	0.006	0.045	1.18
9	hsa‐miR‐520d‐5p	0.0001	0.007	NS	1.21
10	hsa‐miR‐505*	0.0001	0.006	NS	1.15
11	hsa‐miR‐20b*	0.0002	0.008	NS	1.12
12	hsa‐miR‐2355‐3p	0.0002	0.008	NS	1.18
13	hsa‐miR‐7	0.0003	0.010	NS	1.10
14	hsa‐miR‐181b	0.0003	0.010	NS	1.15
15	hsa‐miR‐584	0.0004	0.012	NS	1.12
16	hsa‐miR‐516b	0.0004	0.012	NS	1.17
17	hsa‐miR‐488	0.0006	0.017	NS	1.10
18	hsa‐miR‐877	0.0006	0.016	NS	1.14
19	hsa‐miR‐485‐3p	0.0006	0.016	NS	1.13
20	hsa‐miR‐3614‐3p	0.0006	0.016	NS	1.14
21	hsa‐miR‐4301	0.0007	0.017	NS	1.24
22	hsa‐miR‐361‐3p	0.0010	0.023	NS	0.78
23	hsa‐miR‐1909	0.0010	0.022	NS	1.15
24	hsa‐miR‐3201	0.0010	0.021	NS	1.17
25	hsa‐miR‐181d	0.0011	0.021	NS	1.25
26	hsa‐miR‐934	0.0011	0.020	NS	1.16
27	hsa‐miR‐374b*	0.0011	0.020	NS	1.13
28	hsa‐miR‐551b*	0.0012	0.020	NS	1.17
29	hsa‐miR‐3654	0.0012	0.020	NS	1.16
30	ebv‐miRBART18‐3p	0.0012	0.019	NS	1.15
31	hsa‐miR‐1321	0.0013	0.020	NS	1.15
32	hsa‐miR‐553	0.0013	0.020	NS	1.15
33	hsv2‐miR‐H20	0.0013	0.020	NS	1.12
34	hsa‐miR‐2113	0.0014	0.020	NS	1.13
35	hsa‐miR‐124*	0.0015	0.021	NS	1.22
36	hsa‐miR‐200b	0.0022	0.030	NS	1.47
37	hsa‐miR‐3606	0.0023	0.030	NS	1.11
38	hsa‐miR‐1184	0.0024	0.031	NS	1.22
39	hsa‐miR‐3148	0.0026	0.033	NS	1.18
40	hsa‐let‐7b	0.0027	0.033	NS	1.25
41	hsa‐miR‐3183	0.0030	0.035	NS	1.09
42	ebv‐miRBART17‐3p	0.0031	0.036	NS	0.89
43	hsa‐miR‐630	0.0034	0.038	NS	1.16
44	hsa‐miR‐1469	0.0038	0.042	NS	1.29

NS, not significant.

**Table 3:** Characteristics of the eight miRNAs differing most significantly between NAFLD and non‐NAFLD liver

miRNA	miRBase ID	B^a^	H	S	E	M	No. of pred. targets	Previous functional indications	References
mir‐103a‐2*	MIMAT0009196	+	+	+	+	+	169	–	–
miR‐106b*	MIMAT0004672	+	+	+	+	+	155	Upregulated in ovarian cancer and laryngeal carcinoma	Liu et al. (2014), Lu et al. (2014)
miR‐576‐5p	MIMAT0003241	+	+	+	+	+	4996	Associations with cancers, pertussis, and systemic lupus	Li et al. (2012), Ge et al. (2013), Mairinger et al. (2014), Martinez‐Ramos et al. (2014)
miRPlus‐I137*	–	+	+	+	+/−	+/−	−	–	–
miR‐892a	MIMAT0004907	+	−	−	+/−	+	4302	Upregulated in bile of patients with biliary strictures; Regulator of *CYP1A1*	Choi et al. (2012), Lankisch et al. (2014)
miR‐1282	MIMAT0005940	+	+/−	−	−	+	468	Upregulated by all‐trans retinoic acid in HCC cells	Wang et al. (2013)
miR‐3663‐5p	MIMAT0018084	−	−	−	−	−	167	Upregulated in cutaneous malignant melanoma	Sand et al. (2013)
miR‐3924	MIMAT0018199	−	−	−	−	−	1075	–	–

a Detectable by qPCR in total RNA from the liver biopsies (B), in miRNA preparations from primary human hepatocytes (H), stellate cells (S), sinusoid endothelial cells (E), or in total RNA from primary human monocyte‐derived macrophages (M).

### Predicted targets of the NAFLD‐associated miRNAs

**Figure 3:**
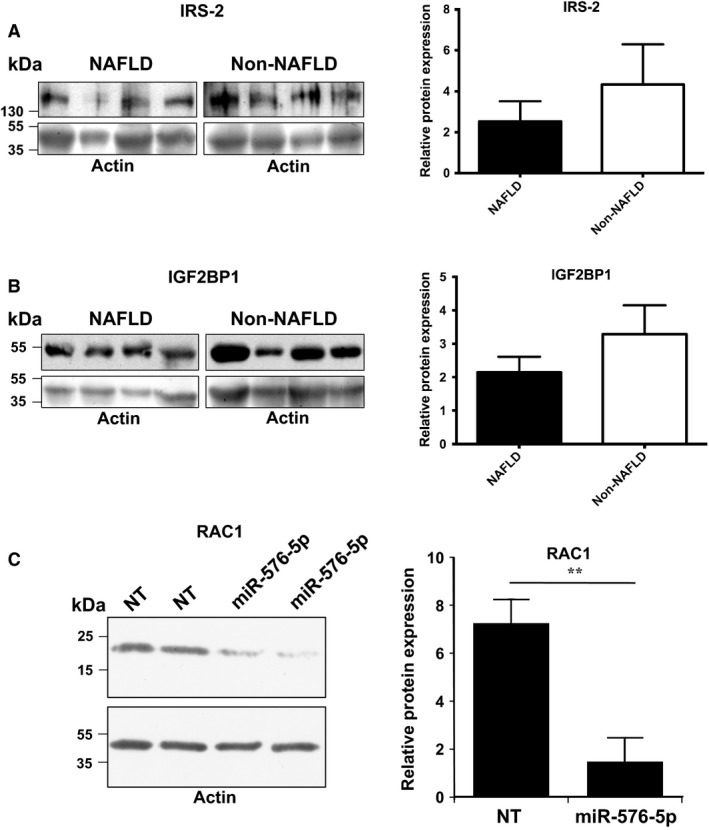
Western analysis of predicted miRNA targets. (A) Analysis of IRS‐2 in NAFLD and non‐NAFLD liver biopsies. Left: Representative blots of 4 NAFLD and 4 non‐NAFLD biopsies. Right: Quantification of IRS‐2 signals relative to β‐actin; NAFLD, *n* = 13, non‐NAFLD, *n* = 11. (B): Analysis of IGF2BP1 in NAFLD and non‐NAFLD liver biopsies. Left: Representative blots of 4 NAFLD and 4 non‐NAFLD biopsies. Right: Quantification of IGF2BP1 signals relative to β‐actin; NAFLD, *n* = 16, non‐NAFLD, *n* = 12. (C) Western analysis of RAC1 protein expression in IHH transfected for 48 h with nontargeting control miRNA (NT) or the miR‐576‐5p mimic (miR‐576‐5p). Right: Quantification of the RAC1 signal relative to β‐actin; *n* = 4. The data represent mean ± SEM, **P < 0.01.

Target mRNAs were predicted for seven of the eight miRNAs differing most significantly between the NAFLD and non‐NAFLD groups (miR‐103a‐2*, miR‐106b, miR‐576‐5p, miR‐892a, miR‐1282, miR‐3663‐5p, miR‐3924), whereas no targets could be predicted for miRPlus‐I137*. The criterium for target identification was consistent prediction by a minimum of three algorithms. The numbers of putative targets varied from 155 (miR‐106b*) to 4996 (miR‐576‐5p) (Table 3). The target gene lists were submitted to the Ingenuity software to identify canonical target pathways. After BH multiple test correction, potential target pathways could be identified for four of the miRNAs, miR‐103a‐2*, miR‐576‐5p, miR‐892a, and miR‐1282. The pathway predicted to be most significantly downregulated by all four miRNAs was “Molecular mechanisms of cancer.” The other target pathways identified repeatedly for the different miRNAs included “PPARα/RXRα (peroxisome proliferator‐regulated receptor‐α/retinoid X receptor‐α) activation,” “PTEN (phosphatase and tensin homologue) signaling,” “PI3K/AKT (phosphoinositide‐3‐kinase/protein kinase B) signaling,” “NF‐κB (nuclear factor kappa B) activation by viruses,” and “IL‐8 (interleukin‐8) signaling.” To investigate whether gene products predicted to be subjected to regulation by several of the miRNAs upregulated in NAFLD are reduced in the liver biopsies, we carried out western analyses with anti‐IRS‐2 (predicted to be targeted by miR‐103a‐2*, miR‐576‐5p, miR‐892a, miR‐1282, and miR‐3924, by a minimum of 3 distinct algorithms) and anti‐IGF2BP1 (predicted to be targeted by miR‐576‐5p, miR‐892a, miR‐1282, miR‐3663‐5p, and miR‐3924). The analysis revealed a tendency of downregulation for both proteins (IRS‐2, mean −42%; IGF2BP1, mean −35%) in the NAFLD as compared to non‐NAFLD biopsies. However, with the number of liver biopsy protein specimens available (non‐NAFLD, *n* = 11–12; NAFLD, *n* = 13–16), the differences did not reach statistical significance (Fig. 3A and B).

### Functional analysis of miR‐576‐5p in hepatocytes

We next selected the miRNA species with the highest number of predicted targets, miR‐576‐5p, for functional analysis in the noncancerous IHH. To study the impact of elevated miRNA levels on hepatocyte gene expression, IHH were transfected with a miR‐576‐5p mimic or a control miRNA, followed by microarray transcriptome analysis. This analysis revealed significant alterations in 478 messages (with stringent criteria, BH corrected *P* < 0.001). Of the mRNAs suppressed upon miR‐576‐5p transfection 47 represented predicted direct targets of the miRNA (Table 4). The messages affected by miR‐576‐5p transfection were next subjected to Ingenuity pathway analysis: The 19 pathways significantly altered (Table 5) contained a total of 233 affected mRNAs, of which 134 were down‐ and 99 upregulated. These pathways included four of the predicted target pathways of miR‐576‐5p: “mTOR signaling,” “Ephrin B signaling,” “Breast cancer regulation by stathmin,” and “Production of nitric oxide and ROS in macrophages.” In these pathways, the predicted direct targets of miR‐576‐5p were *RAC1* (the small GTPase RAC1), *PRKCI* (protein kinase C iota), *GNAS* (stimulatory G‐protein α‐subunit), *CFL2* (cofilin 2), and *ACP1* (low molecular weight phosphotyrosine protein phosphatase) (Table 6). A marked downregulation of RAC1, shared by all the above four pathways, was verified at the protein level by western analysis of transfected IHH (Fig. 3C). For technical reasons, we could not assess the quantity of RAC1 protein in the liver biopsies of subjects with NAFLD: Possibly due to an elevated lipid content of the protein specimens from NAFLD liver biopsies, protein species <30 kDa were poorly detectable on the western filters. In addition to the predicted miR‐576‐5p target pathways, those identified as affected in the Ingenuity analysis included several pathways of interest, such as “Regulation of eIF4 and p70S6K signaling,” pathways of cell–cell junctional signaling, “3‐phosphoinositide synthesis,” and “3‐phosphoinositide degradation.”

**Table 4:** Predicted target mRNAs of miR‐576‐5p suppressed by miR‐576‐5p mimic transfection into IHH

Num bers	mRNA	EntrezGeneID	FC^a^	*P*‐value, BH
1	PTER	9317	−1.46	0.000124475
2	SERBP1	26135	−1.34	0.000147414
3	RNF11	26994	−1.29	0.000279392
4	PHF13	148479	−1.2	0.000343062
5	TOP3A	7156	−1.33	0.000351337
6	AP2A2	161	−1.46	0.000351337
7	CFL2	1073	−1.28	0.000351337
8	TM7SF3	51768	−1.08	0.000421164
9	KLF6	1316	−1.29	0.000425895
10	PRPF4	9128	−1.21	0.000459303
11	GNAS	2778	−1.41	0.000463309
12	KDSR	2531	−1.28	0.00049638
13	ATP11B	23200	−1.31	0.000506341
14	RFK	55312	−1.3	0.000532456
15	TFB2M	64216	−1.09	0.000540098
16	LIPG	9388	−1.77	0.000543642
17	EIF2S2	8894	−1.18	0.000575617
18	STYX	6815	−1.48	0.000579546
19	PIGX	54965	−1.3	0.000593584
20	RAC1	5879	−1.2	0.000662758
21	DISC1	27185	−1.15	0.000688879
22	NIPA1	123606	−1.32	0.000696155
23	UBE2N	7334	−1.2	0.000709105
24	PSMD12	5718	−1.2	0.000713024
25	THEM4	117145	−1.47	0.00073446
26	NDUFA5	4698	−1.16	0.000737784
27	TET2	54790	−1.65	0.000737784
28	EPAS1	2034	−1.26	0.000741408
29	MXRA7	439921	−1.2	0.000741408
30	TIPRL	261726	−1.28	0.000743489
31	MTDH	92140	−1.43	0.00074509
32	PFKFB2	5208	−1.34	0.000760532
33	RCOR1	23186	−1.26	0.000767747
34	NDRG3	57446	−1.48	0.000782116
35	RUNDC1	146923	−1.44	0.000782116
36	VTA1	51534	−1.26	0.000783553
37	CD55	1604	−1.27	0.000783553
38	CYP20A1	57404	−1.25	0.000783553
39	ACP1	52	−1.09	0.000792571
40	DAB2IP	153090	−1.16	0.000805472
41	SLC31A1	1317	−1.44	0.000824846
42	MLH3	27030	−1.23	0.00084937
43	TBRG1	84897	−1.43	0.000867622
44	SLC23A2	9962	−1.32	0.000941428
45	EGR3	1960	−2.64	0.000944807
46	PRKCI	5584	−1.19	0.000952935
47	METTL10	399818	−1.22	0.000971469

a Fold change.

**Table 5:** Canonical Ingenuity pathways significantly affected by miR‐576‐5p transfection into IHH

Pathway	No. of mRNAs down/upregulated	*P*‐value, BH
Regulation of eIF4 and p70S6K Signaling	7/9	0.000021
mTOR Signaling^a^	10/8	0.000021
Epithelial Adherens Junction Signaling	10/4	0.00042
Germ Cell‐Sertoli Cell Junction Signaling	11/3	0.00091
Sertoli Cell‐Sertoli Cell Junction Signaling	9/4	0.0098
D‐myo‐inositol (1,4,5,6)‐Tetrakisphosphate Biosynthesis	4/6	0.018
D‐myo‐inositol (3,4,5,6)‐Tetrakisphosphate Biosynthesis	4/6	0.018
Cdc42 Signaling	5/5	0.026
Remodeling of Epithelial Adherens Junctions	7/0	0.026
D‐myo‐inositol‐5‐phosphate Metabolism	4/6	0.032
3‐phosphoinositide Degradation	4/6	0.032
14‐3‐3‐mediated Signaling	6/3	0.032
Ephrin B Signaling^a^	5/2	0.032
Breast Cancer Regulation by Stathmin1^a^	10/2	0.032
RhoGDI Signaling	9/2	0.041
3‐phosphoinositide Biosynthesis	4/6	0.041
Signaling by Rho Family GTPases	10/3	0.049
Production of NO and ROS in Macrophages^a^	6/5	0.049
EIF2 Signaling	9/19 134/99	0.049

a Predicted target pathways of miR‐576‐5p.

**Table 6:** Constituent genes of the predicted 576‐5p target pathways significantly affected by miRNA transfection into IHH

Canonical pathway	Constituent genes
mTOR Signaling	ULK1,RPS3A,PRKAB2,RHOC,RAC1 a,RPS10,PPP2R5A,ATG13,RPS7,PRKCI^a^,EIF3D,RPS13,EIF4A1,RPS27A,RPS25,EIF3L,RPS24,RPSA
Ephrin B Signaling	VAV2,GNAS^a^,CFL2^a^,GNB2L1,RAC1 a,ACP1^a^,EFNB3
Breast Cancer Regulation by Stathmin1	TUBB3,GNAS^a^,PRKCI^a^,TUBA1A,PPP1R12A,GNB2L1,RAC1 a,PPP1R11,TUBA1C,TUBB,PPP1CA,PPP2R5A
Production of Nitric Oxide and ROS in Macrophages	TUBB3,GNAS^a^,PRKCI^a^,TUBA1A,PPP1R12A,GNB2L1,RAC1 a,PPP1R11,TUBA1C,TUBB,PPP1CA,PPP2R5A

a Predicted direct targets; RAC1 shared by the pathways is underlined.

## Discussion

We performed, on a microarray platform with the largest selection of miRNA probes thus far employed, profiling of liver biopsies from obese human subjects with NAFLD or normal liver histology, selecting the patients at the extreme ends of liver fat content. Differential expression of 44 miRNAs in NAFLD as compared to non‐NAFLD liver was suggested by the microarray analysis, and elevated expression of several of these miRNAs in NAFLD versus non‐NAFLD liver was confirmed by qPCR. In further analysis, we focused on the eight miRNAs differing most significantly between the groups (all identified as upregulated in NAFLD liver). Their function was assessed (1) by prediction and Ingenuity pathway analysis of their putative targets, (2) by investigating their cell type‐specific expression, (3) by western blotting of liver biopsy specimens with antibodies against predicted targets shared by multiple miRNAs, and (4) by studying the impact of miR‐576‐5p, the species with the largest number of predicted targets (4996), on the transcript profile of IHH.

Of the 44 miRNAs identified as differentially expressed, 42 are novel in the context of NAFLD, only two (miR‐181b and miR‐200b, induced in the NAFLD group) being previously associated with NAFLD in humans or in murine models. Cheung et al. (2008) found miR‐181b elevated in advanced NAFLD relative to subjects with normal liver histology. This miRNA was shown to promote the activation of stellate cells and correlated with liver fibrosis in a rat model of liver disease (Zheng et al. 2014). MiR‐200b, a species with tumor suppressor activity, was found upregulated in diet‐induced murine models for NAFLD (Pogribny et al. 2010; Alisi et al. 2011). In addition, Whittaker et al. (2010) identified miR‐181d, upregulated in the present NAFLD group, as a species that reduces lipid droplet formation in cultured hepatocytes. The reported functional implications thus suggest that these miRNAs may impact the progression of NAFLD.

Discovery of a large number of novel NAFLD‐associated miRNAs in our study is likely due to the following factors: (1) Unlike earlier studies, we employed microarrays carrying probes for 1438 human miRNAs, thus covering a majority of the human miRNome. The most extensive miRNA screen of human NAFLD previously conducted employed a platform with probes for 474 human miRNA species (Cheung et al. 2008). (2) We did not limit the study to patients with advanced NAFLD but also included subjects without signs of steatohepatitis. (3) We employed an extreme end strategy, selecting subjects with the lowest and the highest liver fat content. (4) The present subjects were markedly more obese (NAFLD group BMI 49.2 and non‐NAFLD group 46.5) than those investigated in Cheung et al. (2008) (BMIs 35 and 39.5, respectively), and the liver biopsies were withdrawn during bariatric surgery. The degree of obesity and the hypocaloric diet preceding surgery may have modified the hepatic miRNA expression pattern. However, the impact of this has not been investigated. Further studies are thus required to assess how general the present observations are among different groups of patients with NAFLD. The number of miRNAs detected was 299–389, which most likely underestimates the number of hepatic miRNAs. This, together with a moderate group size (*N* = 15) may have caused a failure to detect some differentially expressed miRNAs. We could not validate dysregulation of all miRNAs subjected to qPCR analysis of the liver biopsy RNA specimens. This may be due to either a lower sensitivity or selectivity of the qPCR methology as compared to the microarray, or erroneous signals in the microarray hybridization. Therefore, confirmation of the microarray findings with another, independent approach is necessary before further functional analysis of the now discovered NAFLD‐associated miRNA species. The fold change of upregulation of the miRNAs under detailed investigation was moderate, between 1.11 and 1.20. The upregulation of any individual miRNA is thus unlikely to result in detectable changes in quantity of gene products. However, we envision that such minor changes in a number of miRNAs (in this case up to 42), many of which share some of the same targets, sum up to physiologically important regulatory effects. Evidence supporting this was obtained by western analyses of IRS‐2 and IGF2BP1, targets each shared by five of the miRNAs under study. The mean expression level of both proteins showed a tendency of reduction in the liver of subjects with NAFLD. IRS‐2 was previously shown to be suppressed in NAFLD liver, resulting in enhanced lipogenesis (Taniguchi et al. 2005; Kohjima et al. 2008), and IGF2BP1 is an important protumorigenic factor upregulated in hepatocellular carcinoma (Gutschner et al. 2014). Downregulation of IRS‐2 may thus promote hepatic lipid accumulation, whereas suppression of IGF2BP1 can be envisioned to represent a response protecting the liver from oncogenesis.

The main limitation in miRNA research is accurate prediction of targets (Pasquinelli 2012). To cope with this problem, we employed nine different algorithms and accepted as putative targets only those identified by a minimum of three algorithms. In pathway analysis we focused on those targeted by several of the new miRNAs – such pathways are the most likely candidates to be affected *in vivo* by the altered miRNA expression pattern. The analysis revealed “Molecular mechanisms of cancer” as a major target of several miRNAs. Association of NAFLD with hepatocellular carcinoma is well established (White et al. 2012). Thus, suppression of cancer‐associated genes by the miRNAs could represent a protective response. The other target pathways identified repeatedly included “PPARα/RXRα activation,” “PTEN signaling,” “PI3K/AKT signaling,” “NF‐κB activation by viruses,” and “IL‐8 signaling.” PPARα is a major transcriptional controller of hepatic lipid metabolism (Tyagi et al. 2011), which is downregulated in the liver of subjects with NAFLD or obesity‐related insulin resistance (Kohjima et al. 2007; Pettinelli et al. 2009), putatively favoring lipogenesis over fatty acid oxidation. Moreover, downregulation of PPARα facilitates the activity of hepatic proinflammatory cytokines, expediting transition from steatosis to steatohepatitis (Videla and Pettinelli 2012). Suppression of the PPARα/RXRα pathway by miRNAs may thus promote the progression of liver disease.

The PI3K/AKT pathway is the major effector of insulin action, and PTEN antagonizes AKT, thus negatively regulating insulin signaling (Schultze et al. 2012). Dysregulation of PI3K/AKT and PTEN pathways by abnormal miRNA patterns may thus contribute to the insulin resistance associated with NAFLD (Yki‐Järvinen 2014). The NF‐κB and IL‐8 pathways are triggered by inflammatory stimuli and promote the progression of steatosis toward advanced forms of liver disease (Braunersreuther et al. 2012). Their suppression by miRNAs could represent a protective response. Of the new miRNAs validated to be upregulated in NAFLD, miR‐892a was in cell‐type‐specific expression analysis only detectable in primary macrophages, suggesting that the microarray signal for this species could originate from Kupffer cells. Its elevated expression may thus associate with Kupffer cell activation (Meli et al. 2014). Study of a putative function of miR‐892a in the Kupffer cells of fatty liver is thus warranted.

As a proof‐of‐principle experiment, we investigated the transcriptome of hepatocytes transfected with one of the miRNAs upregulated in NAFLD, miR‐576‐5p, the species with the highest number of predicted targets. The transcripts affected by the miRNA included many species up‐ and not downregulated, and a number of the suppressed mRNAs are not predicted targets of the miRNA. This is consistent with the perception that regulation of gene expression by miRNAs involves networks of both direct and indirect effects and mutual cross‐talk with transcription factor systems (Tsang et al. 2007; Selbach et al. 2008). Moreover, NAFLD involves an array of gene expression changes driven by mechanisms other than miRNA dysregulation (Naik et al. 2013; Mazzoccoli et al. 2014). Thus, effects of a single miRNA *in vitro* can hardly be expected to result in phenotypic changes resembling those in NAFLD liver. Nevertheless, several pathways affected by miR‐576‐5p transfection are linked to the pathogenesis of NAFLD. Of these, “mTOR signaling” is most interesting. mTOR, a serine/threonine kinase subject to regulation by insulin, regulates the prolipogenic transcription factor SREBP1 at multiple levels (Bakan and Laplante 2012), and a route mediated by mTOR and PPARγ promotes hepatic lipogenesis (Li et al. 2014b). The pathways modulated by miR‐576‐5p also included “Regulation of eIF4 and p70S6K signaling,” an effector pathway that controls protein synthesis and is subject to mTOR regulation (Morita et al. 2015) and “3‐phosphoinositide synthesis/degradation” associated with the major target of insulin action, the PI3K signaling route. To summarize, the transcriptome alterations induced by miR‐576‐5p transfection are consistent with a role of this miRNA species as a modulator of insulin signaling and metabolic control in hepatocytes. However, miRNAs not only affect the stability of mRNAs but also their translation (Vasudevan et al. 2007; Selbach et al. 2008). The transcriptome profiling approach employed therefore only captures part of the functional effects of miR‐576‐5p in the transfected cells. In several pathways affected by miR‐576‐5p, a common gene suppressed was *RAC1* (predicted direct target of miR‐576‐5p). The RAC1 protein was markedly reduced by miR‐576‐5p transfection into IHH, providing further evidence that the message is a relevant target of this miRNA. Consistent with the present observations, *RAC1* is reported to be suppressed in NAFLD liver (Baker et al. 2010; Lopez‐Vicario et al. 2014). RAC1 contributes to stimulation of Jun N‐terminal kinase (JNK) by saturated fatty acids, promoting their lipotoxicity (Sharma et al. 2012). Thus, the suppression of *RAC1* by miR‐576‐5p may act protective against progression of liver disease. To summarize, analysis of the predicted target pathways of the miRNAs under investigation and ones modified by transfection with miR‐576‐5p mimics suggested alterations some of which may promote the progression of NAFLD (e.g., suppression “PPARα/RXRα activation” and “PI3K/AKT signaling”), whereas others may rather protect the liver from the pathogenic processes associated with this disease (e.g., suppression of “NF‐κB activation by viruses,” “IL‐8 signaling,” and “Production of nitric oxide and ROS in macrophages”). Due to the limited number of subjects, the present data do not allow us to analyze how the observed changes in miRNA expression may be modified upon progression of NAFLD from simple steatosis to advanced liver injury.

In conclusion, our profiling data suggest dysregulation of 44 miRNAs in fatty liver, 42 of which are novel in the context of NAFLD. The eight miRNAs most significantly upregulated in NAFLD were subjected to further study. The work demonstrates that by applying a novel type of study set‐up and a broad‐coverage array platform one can reveal a wealth of previously undiscovered miRNA dysregulation in metabolic disease.

## Conflict of Interest

The authors declare that they have no conflicts of interest, financial or otherwise, to disclose.
